# Lectures on the Diseases of the Urinary Organs

**Published:** 1834-01-01

**Authors:** 


*Lectures on the Diseases of the Urinary Organs.* By B. C. Brodie, F.R.S., Serjeant Surgeon to the King, and Surgeon to St. George's Hospital.—8vo. pp. 306. London, 1833.

Mr. Brodie's appearance in print is less frequent than we might be justified in expecting from his known zeal and opportunities. He has never written without adding to his reputation; and he has certainly increased those stores which none more than English surgeons have contributed to enrich. The next best act to devoting his attention to the subject of these lectures, is recalling them from their fugitive form, and investing them with additional matter in a comely octavo. In his advertisement he well describes the distinguishing feature of all his writings: "I have endeavoured to record what I have seen, with accuracy and fidelity, making it my especial object to explain and illustrate those circumstances which were to myself a source of doubt and difficulty in the earlier part of my practice."

Incessant devotion to a harassing occupation leaves but few opportunities for cultivating the graces of composition: indeed, it is too often to be regretted that excellent works suffer an abatement of their value from the uninviting character of their style. Mr. Brodie's writing, if it challenge not our admiration for its eloquence, is never tedious, and certainly is not deficient in the perspicuity essential to the description of subjects for the most part practical.

In the long catalogue of human ills, there is not a more interesting and important class than that which forms the subject of these lectures. Very few will dissent from our opinion, that the present volume is the best on the subject; not that it contains much information unknown to practitioners familiar with this branch of surgery, but that it comprehends and condenses nearly *all* that is known, thus making the knowledge more available by rendering it easy of access. It were unmerited praise, unmeaning flattery—Mr. Brodie can well afford to dispense with both,—to commend this work for its originality. In these days so closely does one competitor tread on the kibes of another, that we rarely see the possessors of activity and intelligence greatly distancing each other, and then only in pursuits which are more abstract than practical; and certainly this cannot be predicated of the treatment of urethral, prostatic, and vesical diseases.

The following observations are clear, judicious, and instructive:

"The treatment of fistulæ in perineo, or of those fistulæ which open externally on the scrotum, or nates, or elsewhere, communicating with the urethra behind the stricture, is in most instances very simple. You are not to be misled, by the resemblance in the name, into the belief that these fistulæ require any treatment corresponding to that which is required for fistulæ in ano. The latter are formed among muscular structures, and for the most part in the substance of the sphincter muscle. A fistula in ano requires to be freely laid open, because it is the action of the muscular fibres over it and under it that prevents it healing. The division of the muscular fibres sets them at liberty, and places the fistula under the same circumstances with an ordinary sore. But a fistula in perineo is prevented from healing, not by the action of muscles, but by the urine flowing through it. You will put your patient to unnecessary pain by laying it open. The urine will flow through it still, and, in fact, in more abundant quantity. The wound will heal to a certain point, and then the patient will be in the same state as he was in before. Dilate the stricture, let the urethra be restored to its natural diameter, and as soon as the urine passes freely through the natural passage it will cease to flow through the artificial one; and this being accomplished, the fistula will immediately heal. Sometimes it will heal before the dilatation of the stricture is completed, when the cure of it is only half performed. In other cases the healing of the fistula will be gradual, and it will be necessary to persevere in the occasional use of the bougie for many months before it is completely closed. If the fistula should not heal under this negative treatment, you may resort to other methods. Let the patient remain for three weeks in bed, with the gum catheter constantly retained in the urethra and bladder. This will sometimes succeed, but it will at other times fail. It may fail, 1st, when the opening by which the fistula communicates with the urethra is unusually large; 2dly, where the urine, instead of passing through the catheter, flows by the side of it; and, 3dly, where the instrument brings on an abundant suppuration of the urethra; in which case the purulent discharge finds its way into the fistula, and prevents its healing as much as it would be prevented by the contact of the urine. When then this method fails, or when your patient finds it impossible to make that sacrifice of time, and submit to that degree of confinement, which it requires, you may instruct him in the use of the catheter, and advise him, for some time to come, never to void his urine by his own efforts, but to draw it off by the catheter. You may also stimulate the bottom of the sinus by the occasional introduction of a small piece of the nitrate of silver: at the same time that you retard the healing of the orifice of the sinus, by lightly touching it once in a week or fortnight with the caustic potass. The reason for applying the caustic potass is as follows: the external orifice of the fistula is always more inclined to heal than the bottom of it towards the urethra. If you stimulate the whole of the fistula with the nitrate of silver, the orifice of it is likely to close prematurely, that is, before it is healed at the bottom. The necessary consequence of this is another abscess and another discharge of matter. By applying the caustic potass to the external orifice you prevent this from healing, while the application of the nitrate of silver promotes the growth of granulations within, and the cicatrisation of the more deep-seated part of the fistula." (P. 63.)

Mr. Brodie has before especially directed the attention of his brethren to some of the Protean forms of hysteria simulating local diseases.

"The paralytic affection of the bladder, which occurs in hysterical females, is of a peculiar kind, and deserves a separate consideration. It appears to me that the symptoms are to be traced to a still higher source than in ordinary cases of paralysis; that, in the first instance, it is not that the nerves are rendered incapable of conveying the stimulus of volition, but that the effort of volition is itself wanting; and this corresponds with what is observed in cases of loss of voice, and in many other diseases connected with hysteria. As the distension of the bladder increases, the patient begins to be uneasy, and at last suffers actual pain; and as soon as this happens, the volition is exercised as usual, and the bladder begins to expel its contents.

"Thus, if the bladder be not relieved artificially, by the introduction of the catheter, the hysterical retention of urine is usually of short duration. If, however, the catheter be had recourse to, the natural cure is prevented, and the existence of the disease may be prolonged for an indefinite period of time, for weeks, or even for months. The general rule to be observed in the treatment of these cases is to interfere but little. You may administer an active aperient, or an assafœtida enema, or you may give assafœtida by the mouth, but you should avoid using the catheter. This general rule, however, is not without its exceptions. In a few of these cases, where the bladder has been very much distended, in consequence of this over-distension it loses its power of contraction, and, even though the patient strains to make water, no urine flows. Under these circumstances, it is evident that artificial relief is necessary; and if it be not afforded, more than simple inconvenience may be the result. A young woman was admitted into St. George's Hospital, in November, 1814, labouring under a train of symptoms which I believe to have been connected with the same condition of the nervous system as that which produces the phenomena of hysteria. I should be wandering from my subject, if I were to relate to you all the circumstances of this interesting and important case. It is sufficient for our present purpose that you should be informed that one of the symptoms was a retention of urine, which had been long neglected, and which existed to such an extent that forty ounces of urine were drawn off by the catheter; and that the patient ultimately died. In my notes, I find the following account of the appearances which the bladder presented in the post-mortem examination: 'It was of a very large size, as if it had been for a long time unusually dilated. It was throughout of a dark colour, almost black. There were only some slight vestiges of its natural structure left; the muscular fibres being very much wasted, and the internal membrane presenting the appearance of a very thin film, which was readily separated from the parts below. The dark colour of the bladder did not seem to arise from mortification, since there was neither fœtor, nor any other mark of putrefaction.' The state of the bladder was indeed very peculiar, not resembling any thing which has fallen under my observation either before or since." (P. 78.)

When the opinions of those who enjoy ample opportunities of judging are at variance with principles generally received, and as extensively justified, it is incumbent on the propounders of a new doctrine to give some good account of the reasons of their dissent from the old. This, Mr. B. has not done when he denies the general inexpediency of bloodletting in inflammation of the bladder, and alleges debility as the prohibitory cause. This is a stronger objection to *general* bloodletting than to local depletion, which, we believe, is defensible by the success of the majority of the profession.

For general utility, because the knowledge is most frequently needed, the remarks on Sand in the Urine are incontestibly the best in the book. Uric acid, first well described by Scheele, and now oftener called lithic acid, was supposed to be held pure in solution by the urine. It is, however, nearly insoluble when pure, but very soluble when in combination with ammonia: it forms lithate of ammonia, which, though nominally a neutral salt, still reddens litmus paper. This is the common deposit of the urine of dyspeptics. If to healthy urine be added any matter for which ammonia has a stronger affinity than for lithic acid, the lithate of ammonia is no longer precipitated, but in its place pure lithic acid. When this decomposition happens in the body, by the presence of another acid, (which Dr. Prout believes to be frequently the muriatic,) the consequence is a deposit of red sand. To the deposition of this sand nothing contributes more than errors in diet, intemperance, and indolence. The red sand of urine, which is composed of the crystals of lithic acid, in combination with soda, constitutes the chalk-stones with which the bursæ and cellular membrane of gouty patients are afflicted. The formation of red sand may be prevented by the administration of potass, soda, lime-water, ammonia, and magnesia, according to circumstances.

"If the lithic acid is deposited in small quantity, and the bowels are too much relaxed, (which, however, rarely happens in these cases,) lime-water may be useful. In persons of weak bodily powers, who may be supposed to require cordial and stimulating remedies, you may exhibit ammonia. Dr. Prout recommends the carbonate of potash in preference to the carbonate of soda, for the following reason; that the soda, under certain circumstances, will enter into combination with the lithic acid, forming an insoluble salt, as bad as the lithic acid itself, whereas the lithate of potash is perfectly soluble; and if this combination takes place, it will pass off dissolved in the urine. On the whole, magnesia, as recommended by Professor Brande, is preferable to the rest. Being in itself insoluble, it cannot enter the circulation except it has first become combined with acid in the stomach or intestine; and hence it does not pass out of the system so soon as the alkalies. The dose of all these remedies must vary according to the circumstances. You may give of the pure magnesia from ten grains to two scruples daily, and of the others in proportion.

"I have mentioned the carbonates of potash, soda, and ammonia, as these agree better with the stomach, and therefore are more proper to be employed than the pure alkalies. The carbonic acid does not interfere with their medicinal effects. There is a remarkable difference in the effects produced on these disorders by the salts which contain a mineral, and those which contain a vegetable acid. The sulphates, muriates, nitrates, are of no avail; but the tartrate of potash, the tartarized soda, the common saline draught composed of citric acid and potash, all produce the same effect as the pure alkalies, or as the alkalies combined with carbonic acid. This remarkable circumstance was first noticed by Sir Gilbert Blane. Sir Gilbert has also recommended a very efficient method of exhibiting the carbonate of potash in these cases, by giving it in a saline draught with an excess of alkali.

"I have said that different doses of the alkaline remedies will be required in different instances. Indeed, a good deal of care is generally necessary to adjust the dose to the peculiar circumstances of the individual case. If you give too little of the alkali, the result is not obtained, and the lithic acid is still deposited, although in smaller quantity. If you give too much, you not only prevent the formation of the red sand, but you render the urine alkaline, and a white sand (the triple phosphate of ammonia and magnesia) is deposited in its place. If magnesia is taken in a larger quantity than is necessary to neutralise the acid generated in the stomach, the patient is liable to the formation of magnesian calculi in the intestines. These last are composed of the magnesia mechanically blended with the fæces and intestinal mucus. They are not uncommon in these times, when so many individuals are in the habit of taking magnesia in a careless and profuse manner. I have in several instances known a person to suffer a good deal of distress from such a calculus being lodged in the rectum. But cases have occurred, in which the accumulation of magnesia in the intestine has taken place to a very great extent. Mr. Wilson examined the body of a patient, in whom, if I recollect rightly, many pounds of magnesia were found collected in the colon above a contracted part of the rectum.

"In the exhibition of alkaline remedies, then, you must make each case the subject of a distinct experiment; and that the experiment may be more properly conducted, you must, if possible, make the patient enter into your views, that he may assist your practice by his own observations. You should be provided with paper, coloured blue by an infusion of litmus; and also with the same paper, slightly reddened by immersion in a very weak acid. Healthy urine ought to turn the blue litmus paper a little red, and you ought not to give alkaline remedies in such a dose as to destroy this property altogether; still less ought you to render the urine alkaline. If the urine turns the red paper blue, the patient is in danger of suffering from a deposition of the phosphates, and the alkalies must be given in smaller quantity.

"It is to be further observed, that the time when the urine is most acid, and when the alkalies are most required, is after the principal meal, that is, after dinner. The alkalies are not indeed to be given immediately after dinner, for then they are likely to interfere with digestion, but three or four hours afterwards. In some cases it is better for the patient to defer taking his medicine until he wakes accidentally in the middle of the night. In many instances, a single dose of magnesia daily, and that at bed-time, is all that is required; while, in others, it should be exhibited in the middle of the day also.

"But it may truly be observed that this is not striking at the root of the disorder. Alkalies prevent the formation of red sand while they are being taken, but they do not prevent it being formed again as soon as they are left off. The patient cannot well take them for ever; and something further, therefore, is required. When he suffers from costiveness, purgatives must be exhibited; and even in those cases in which the bowels are not particularly torpid, purgatives are useful. The mercurial purgatives are, on the whole, to be preferred. A blue pill may be administered every night, with a draught of infusion of senna and tartrate of potash every fourth morning; or a calomel pill may be given once or twice in a week, at bed-time, and the senna draught on the following morning. When the disease is connected with gout, the patient may take the colchicum with great advantage. In the first instance, twenty drops of the vinum colchici may be administered twice or three times daily; afterwards, a draught of infusion of senna, with a saline purgative, and forty or forty-five drops of the vinum colchici, may be given occasionally in the morning.

"But more, after all, is to be effected by attention to diet and mode of living, than by medicine. Is the patient a great eater, pampering his appetite by a variety of dishes, and thus exciting himself to swallow more food than his stomach can readily digest? let him make his dinner on a single dish, and eat of that in moderate quantity. Let him also incline to a diet of vegetable rather than one of animal food; avoiding, however, undressed vegetables, and especially those which are acid or acescent, as salad, oranges, and apples. Does he commit excesses in drinking? let him leave off fermented liquors altogether, or take them only in small quantity; and in particular let him avoid such fermented liquors as, from the sugar which remains unfermented in them, are liable to become acid in the stomach, or which are acid already. The French white wines are injurious in these cases, especially champagne; but none of them are worse than our own English liquor called punch." (P. 152.)

Mr. Brodie believes that one of the most frequent causes of death after lithotomy is the too free division of the prostate gland, extending into the loose cellular tissue. To us, and to others, it does not appear that this opinion is entitled to, or will receive, general assent. The infiltration, abscesses, and sloughing, which follow this operation too frequently, are obviously enough the cause of death; but whether these evils are the commonest results of the too free division of the prostate gland, is more than doubtful. Many eminent authorities, backed by the largest experience of its benefits, recommend a very free incision in this part, of which Mr. B. advises a more niggard division.

The patrons (though few they be) of the gorget may perhaps find, in the views of our author, something like a reason for retaining it, and a probable explanation of its remarkable success in the hands of some operators. We shall be pardoned for extracting Mr. Brodie's picture of the symptoms following too free an incision of the prostate.

"The symptoms which arise in these cases are not well marked in the first instance. There is some heat of skin, and generally an absence of perspiration; there is usually an abundant flow of urine through the wound; the pulse, as to frequency, is somewhat above the natural standard; and the patient, although free from suffering, has no disposition to sleep. This state of things continues for twenty-four, or even for forty-eight hours after the operation; then the more characteristic and alarming symptoms shew themselves; the pulse becomes more frequent, rising to ninety, one hundred, and at last to 140 in a minute; the heat of skin becomes still greater; the tongue dry; the countenance anxious. Afterwards, as you count the pulse, you find every now and then a beat weaker than the rest; and then there are complete intermissions. At first the intermissions are not more than one or two in a minute; by degrees they become more frequent, until they occur every third or fourth beat. There is an occasional hiccough; the patient complains of some degree of tenderness in the lower part of the abdomen, especially in the left groin; the belly becomes tympanitic, that is, the stomach and intestines are filled with air; the distension of the belly increases; the hiccoughs are more frequent; the pulse continuing to intermit, becomes weak and fluttering. In some instances the patient retains his understanding even to the last; while in others he falls into a state of low delirium previous to death. Occasionally, in the progress of such a case, the patient has a severe rigor, and sometimes he complains of a pain in the loins. Where these symptoms begin at an early period, he may die within forty-eight hours from the time of the operation; but in other cases, death may not take place for four or five days, or even for a week. On dissection, you find the cellular membrane round the neck of the bladder, and between the prostate and the rectum, bearing marks of inflammation, infiltrated with lymph and serum, and to a greater or less extent converted into a slough. If death has taken place at an early period, the intestines are found distended with air, and there is a very slight effusion of serum in that part of the peritoneum which descends into the pelvis. But if the patient has laboured under these symptoms for many days before he dies, the peritoneum, where it is reflected from the bladder to the rectum, is seen of a darker colour than natural, and encrusted with lymph; and at a still later period there is the appearance of inflammation, to a greater or less extent, throughout the peritoneum generally. But the peritoneal inflammation is evidently not the primary disease: it is the inflammation and sloughing of the cellular membrane of the pelvis which has induced inflammation of the adjoining portion of that membrane. Something also is to be attributed to the tympanitic distension of the intestines, which, if continued for a considerable time, is always liable to be attended with tenderness of the abdomen, and some degree of peritoneal inflammation.

"It is important that you should not fall into the error of regarding such cases as I have just described as cases of simple peritoneal inflammation, for the remedies which would be useful in the latter case are injurious here. The abstraction of blood, even the operation of an active purgative, will cause the patient to sink more rapidly, tending only to hasten his death. The proper system to be pursued is the opposite to that of depletion. The patient should take such nutriment as his stomach is capable of digesting. The bowels may be kept open by injections, or by the exhibition of some very gentle purgative, and ammonia, wine, and brandy, are to be administered, when the state of the general system indicates that stimulants are necessary.

"Under this kind of treatment I have certainly known two children to recover, who were affected in the manner which I have described. In one of the cases to which I allude, an abscess formed in the neighbourhood of the neck of the bladder, which burst into the wound, and then the symptoms subsided. In the other a slough separated into the rectum, and a fistulous communication remained afterwards between that bowel and the neck of the bladder; but it was of a small size, and productive of no serious inconvenience. In adults the chance of recovery is, at any rate, much smaller than in children. Can anything be done for their assistance in the way of local treatment? Let us consider how it is that the dangerous symptoms arise. There is suppuration and sloughing of the cellular membrane round the neck of the bladder, and the constitution is disturbed, as it is in a case of carbuncle; or, what is still more analogous, as it is in those cases in which there is sloughing of the cellular membrane of the scrotum, in consequence of the effusion of urine arising from the rupture of the urethra behind a stricture. And, in these cases, what is the practice recommended? Do we not divide the soft parts freely over the sloughing cellular membrane: and is not this operation productive of the most signal benefit? Is it possible to resort to any practice corresponding to this, in the cases now under our consideration? There is only one way in which this can be accomplished, namely, by laying the sloughing abscess open into the rectum. I made this experiment in one instance, and I will tell you the result. In September, 1825, I operated on a patient, a man between fifty and sixty years of age, labouring under stone in the bladder, in St. George's Hospital. The stone was extracted without the smallest difficulty: but I performed the operation with what is called Mr. Blizard's lithotomy-knife. This is a long, narrow, straight, probe-pointed bistoury; and you must cut with it laterally, in order that you may divide the prostate, so that it is difficult to determine the exact extent of the incision. Immediately after the operation I had some misgivings, and was led to fear that I had made the incision to such an extent as to penetrate beyond the boundaries of the prostate. At first, indeed, the patient seemed to be going on as well as possible; but, in about forty-eight hours from the time of the operation, some unfavourable symptoms began to shew themselves. On the third day after the operation the countenance was anxious, the skin hot, and the pulse occasionally intermitted. On the following day (the fourth) the pulse intermitted once in fifteen beats; the skin was hot and dry, and the abdomen began to be tense and swollen. I could not doubt that those symptoms existed which I had known to be the precursors of death in some other cases. Under these circumstances, with the concurrence of my colleagues, I performed the operation which I am about to describe. I introduced the forefinger of the left hand into the rectum. I then passed a probe-pointed curved bistoury into the wound, and quite to its farthest extremity on the left side of the neck of the bladder. The probe point having been felt through the tunics of the rectum, I pushed it carefully through them, and, drawing it downwards, divided the lower part of the rectum, sphincter and all. Thus the wound and the rectum were laid into each other. Little or no hemorrhage followed. The relief was immediate. In five minutes after the operation the intermissions of the pulse had diminished from one in fifteen to one in fifty beats. In an hour it did not intermit at all. During the two following days the patient appeared quite well; the pulse was regular, between seventy and eighty in a minute. On the next day there was a slight recurrence of the intermissions of the pulse, but it subsided on the exhibition of some brandy and ammonia. After this there was a progressive amendment; the pulse, however, continuing to beat between eighty and ninety in a minute for the two or three following weeks. After about a month the wound in the rectum began to contract, and the urine to flow by the natural passage; and in another fortnight the patient went into the country, nearly the whole of the urine at this time flowing by the urethra." (P. 271.)

It may reasonably be doubted whether there are not equally important, and more frequently occurring, causes of failure after lithotomy than the "too free incision;" *e.g.* the unavoidable violence essential to the removal of large stones; also that inaptitude of the system to suffer with impunity a disturbing cause of such magnitude as this formidable operation. It must further be observed, that the "too free divisions" of the prostate can hardly help taking place in a much larger number of cases than those in which death occurs. On a review of these considerations, a doubt will present itself whether the too free division be more than a sufficient cause, but of unfrequent occurrence. The occasion of the fatal event of many lithotomic operations has not yet been explained, because not well understood; and assuredly the point can never be determined by the experience of a single individual. Something like a distrust of the soundness of his own reasons is contained in Mr. Brodie's mention of the recto-vesical operation, which he justly prefers to the high operation.

"Here the parts which afford the chief resistance to the extraction of a large stone are divided; and, although the incision of the neck of the bladder extends beyond the boundaries of the prostate, the ill consequences arising from the escape of urine into the cellular membrane are likely to be in great measure obviated, in consequence of the free opening which has been made into the rectum." (P. 293.)

We agree in the opinion of many eminent surgeons, that the blame fixed upon a free internal incision in the lateral operation, may be fairly affiliated on the narrow limits of the external wound; and this with more justice, because it is absurd to suppose that, even when we do not exceed the limits prescribed by the author, we escape the possibility, or the chance of infiltration of urine, and its consequences. Some there are who would without scruple accuse Mr. Brodie of heresy for his approbation of the blunt gorget, and the preference he gives to "splitting" the portion of the prostate left undivided by the knife. On some cardinal points of this operation there exists considerable difference, both in the opinions and the experience of men of equal ability; and we believe the discordance cannot be reconciled by resorting to any one of the grounds of dissent, but only by a reference to the whole.

Of the lithontriptic operation, if he does not speak to its prejudice, his praises are certainly lukewarm: its successful application in appropriate cases will warrant a more animated eulogium than our author's.

Our estimate of the value of these lectures may be inferred from our copious quotations. It would be a comparatively feeble encomium to say that they ought to be in the hands of every professional man; we must go much farther, and declare that they are worthy of the European reputation of Mr. Brodie.

