# Errata

**Published:** 1834-04-01

**Authors:** 


### ERRATA.

In last Number, p. 300, line 18, for "inexpediency," read "expediency."

